# Supplementary material for: Caspase-1 cleaves Bid to release mitochondrial SMAC and drive secondary necrosis in the absence of GSDMD
Source: Life Sci Alliance. 2020 Apr 28;3(6):e202000735. doi: 10.26508/lsa.202000735 (PMC7190276; doi:10.26508/lsa.202000735)

Full Immunoblot of Fig. S4D

siRNA Casp-3		x			x
siRNA Casp-7			x		x
siRNA Casp-8				x	

α-Casp-3
(CST 9662S)
1:1000 in TBST-T
5% milk o/n
1min Pico substrate
(Thermo Fisher)

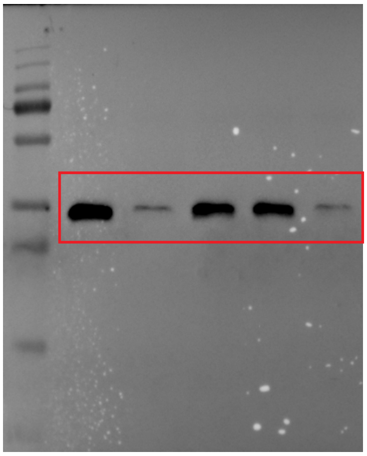

α-Casp-7
(CST 9492S)
1:1000 in TBST-T
5% milk
o/n
2min Pico substrate
(Thermo Fisher)

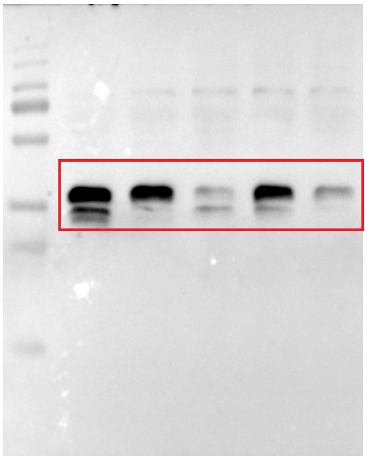

α-Casp-8
(CST 4927S)
1:1000 in TBST-T
5% milk
o/n
3min Femto2 substrate
(Thermo Fisher)

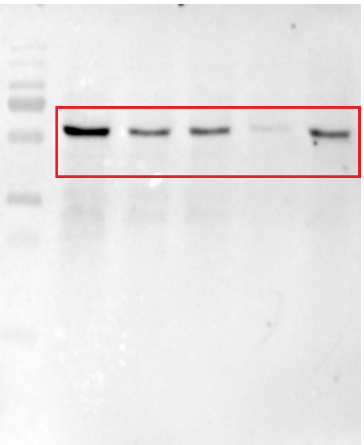

α-Tubulin (ab40742)
1:1000 in TBST-T
5% milk
o/n
3s Pico substrate
(Thermo Fisher)

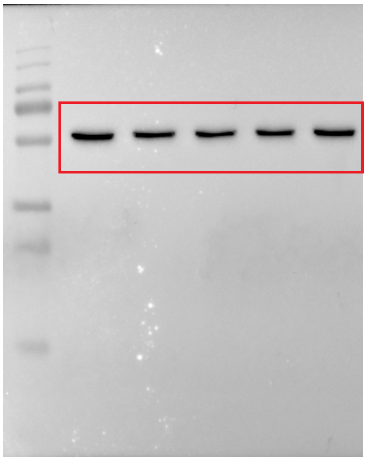

Supplement: Supplementary file 1 [file LSA-2020-00735_SdataFS4.pdf]
